# Discussions on the generalization of HybridSP on more equivalent benchmarks

**DOI:** 10.1093/bib/bbag194

**Published:** 2026-04-25

**Authors:** Zhihao Wang, Sheng Wang, Jingjing Guo, Yuguang Mu, Xiangdong Liu, Liangzhen Zheng, Weifeng Li

**Affiliations:** School of Physics, Shandong University, 27 Shanda Nan Road, Jinan, Shandong Province 250100, China; Shanghai Zelixir Biotech Co. Ltd., 298 Xiangke Road Shanghai 201210, China; Centre for Artificial Intelligence Driven Drug Discovery, Faculty of Applied Science, Macao Polytechnic University, R. de Luís Gonzaga Gomes, Macao 999078, China; School of Biological Sciences, Nanyang Technological University, 50 Nanyang Avenue, 639798, Singapore; School of Physics, Shandong University, 27 Shanda Nan Road, Jinan, Shandong Province 250100, China; Shanghai Zelixir Biotech Co. Ltd., 298 Xiangke Road Shanghai 201210, China; School of Physics, Shandong University, 27 Shanda Nan Road, Jinan, Shandong Province 250100, China

**Keywords:** statistical potential, scoring function, protein-ligand interaction

## Abstract

We thank the reviewers for their thoughtful comments and we have carefully addressed the concerns raised. In our response, we clarify key methodological aspects and provide additional analyses to improve the transparency and rigor of our work. Importantly, we further evaluate the model on more diverse and fair benchmarks beyond the original setting, demonstrating its robustness and generalization ability. These additions strengthen the overall conclusions and provide a more comprehensive assessment of HybridSP.

We thank Marcio and colleagues for their careful reading of our work and for their constructive comments. We appreciate their thoughtful feedback and the recognition of our study [1]. In the following, we provide detailed responses to the concerns raised and clarify several methodological aspects of our work.

## Parameter tuning and benchmark selection

At present, there are several widely accepted virtual screening benchmarks that are considered without bias. However, these datasets are substantially larger in scale. Using them to tune the weighting coefficients would significantly increase the computational cost and reduce efficiency. For this reason, we selected CASF-2016 [2], a compact and widely recognized benchmark, as the criterion for model selection. We acknowledge that tuning on larger and more rigorous benchmark sets may be desirable in the future, but this lies beyond the scope of the present study. As discussed, because tuning and evaluation on the same benchmark may introduce potential data leakage, we further assessed the generalization capability of our method on independent datasets, including PoseBusters [3] and Unbias-v2019 [4] for docking, as well as DUD-E [5], DUD-AD [6], and P450 for virtual screening. The results consistently demonstrate that our algorithm does possess strong generalization capability.

## Comparability between scoring functions and full-pipeline models

Scoring-function-based methods follow a “dock-then-score” paradigm, in which candidate binding poses generated by docking software are rescored and ranked to identify the most plausible protein-ligand complex. In contrast, co-folding models such as AlphaFold3 integrate pose generation and evaluation into a single framework, where metrics such as pLDDT can also be interpreted as a form of confidence-based scoring [7]. Despite these methodological differences, the ultimate objective of both approaches is the same: to identify the most plausible complex structure. For dock-then-score methods, since the pose generation procedure is fixed, the task effectively reduces to evaluating the quality of the scoring function. In contrast, when co-folding models are compared with scoring functions, the evaluation inherently reflects the combined performance of pose generation and scoring. We agree that these comparisons do not measure identical aspects of model performance. Rather than being contradictory, they correspond to different evaluation perspectives: scoring-function comparisons isolate ranking ability, whereas comparisons involving co-folding models reflect end-to-end structure prediction quality. Notably, the AlphaFold3 paper includes comparisons with docking-based approaches such as AutoDock Vina, where poses are similarly selected based on scoring [7]. In this sense, such cross-paradigm comparisons are already part of current benchmarking practices. We also agree that a stratified analysis distinguishing scoring functions from full-pipeline models would provide additional clarity. In particular, metrics beyond Root Mean Square Deviation-based (RMSD-based) success rates, such as side-chain realism or atomic clashes, could offer a more comprehensive evaluation. However, as the primary objective of this work is to assess the performance of different types of scoring functions, we focus on success rate as a consistent and widely used metric under a unified docking setup. Developing fair and systematic comparison frameworks across different pose-generation paradigms is an important direction, which we would be interested in exploring in future work.

## Performance on challenging datasets

Both deep learning methods and HybridSP exhibit performance degradation on the DUD-AD and P450 datasets. Nevertheless, in some cases where the EFs are low, HybridSP still selects top-ranked poses that contain energetically favorable polar interactions (see Figs S2 and S4 in the Supplementary Material 1 of our original paper), which is consistent with the human expert understanding of a “good binding pose” [8, 9]. In the free energy perturbation (FEP) dataset, HybridSP performs worse than mature FEP-based methods, as expected. Statistical potentials infer effective interactions based on the frequency distribution of experimental structures; however, they typically represent a simplified approximation of the potential of mean force. In contrast, the FEP method relies on extensive molecular dynamics simulations with explicit solvent models, allowing for the rigorous capture of both enthalpic and entropic contributions along a defined thermodynamic path. Consequently, the FEP approach is grounded in a more robust statistical mechanics framework, making it the gold standard for accurate free energy calculations. Nevertheless, a correlation coefficient above 0.5, together with a clear and interpretable mechanism, indicates that HybridSP can also serve as an efficient and effective method for large-scale screening of lead compounds.

## Additional evidence for generalization capability

Additional evidence for generalization was obtained through multiple complementary evaluations. All results were gathered in the supplemental materials. We analyzed protein and ligand overlap and observed that performance decreases under strict similarity cutoffs, largely due to reduced data size; however, models trained on larger datasets consistently perform better, highlighting the importance of data coverage. Cross-docking experiments on the 3D-Disco dataset [10] show that HybridSP achieves accuracy comparable to RTMScore and significantly outperforms Vina, with top-5 success rates approaching the theoretical upper bound, indicating reliable identification of near-native poses. Furthermore, semi-prospective screening on CBL-B [11] and CHD1 [12] using large-scale, highly imbalanced datasets (up to 1:500 positive-to-negative ratio) demonstrates strong enrichment performance, with true positives highly ranked and most negatives effectively filtered out. Collectively, these results confirm that HybridSP maintains robust and transferable performance across diverse and realistic scenarios.

## The epistemological status of affinity weighting

The affinity-weighting scheme in HybridSP does not convert the model into a supervised learning framework. Traditional knowledge-based statistical potentials are also not “purely unsupervised” in the machine-learning sense; rather, they are derived from observed structural distributions and transformed into pseudo-free-energy potentials through the inverse Boltzmann relation. In this work, experimental binding affinity is used only as a reweighting factor to correct sampling bias in the statistical distributions, rather than as a prediction target or supervision signal. Therefore, HybridSP remains a knowledge-based statistical potential. The affinity information serves as a physically motivated correction to the potential function, improving the estimation of underlying statistical biases, rather than training a supervised affinity predictor. Accordingly, this does not complicate a straightforward comparison with truly unsupervised statistical potentials.

We are grateful for the insightful comments, which have helped improve the clarity and rigor of our manuscript. We have addressed all the concerns raised and revised the manuscript accordingly. We hope that our responses satisfactorily resolve the issues and further strengthen the presentation of our work.

## Supplementary Material

bbag194_supplementary_materials

## Data Availability

The data is available at https://github.com/zelixirSH/HybridSP.
