# Supplementary material for: Discussions on the generalization of HybridSP on more equivalent benchmarks
Source: Brief Bioinform. 2026 Apr 25;27(2):bbag194. doi: 10.1093/bib/bbag194 (PMC13109047; doi:10.1093/bib/bbag194)
Supplement: bbag194_supplementary_materials [file bbag194_supplementary_materials.pdf]

# Supplementary Materials

Zhihao Wang[1], Sheng Wang[2], Jingjing Guo[3], Yuguang Mu[4], Xiangdong Liu[1], Liangzhen Zheng[2,*], Weifeng Li[1,*]

[1]School of Physics, Shandong University, 27 Shanda Nan Road, 250100 Jinan, Shandong Province, China

[2]Shanghai Zelixir Biotech Co. Ltd., 298 Xiangke Road, 201210 Shanghai, China

[3]Centre for Artificial Intelligence Driven Drug Discovery, Faculty of Applied Science, Macao Polytechnic University, R. de Luís Gonzaga Gomes, 999078 Macao, China

[4]School of Biological Sciences, Nanyang Technological University, 50 Nanyang Avenue, 639798, Singapore

*Corresponding authors. Liangzhen Zheng, Shanghai Zelixir Biotech Co. Ltd, Shanghai 201210, China, E-mail: zhenglz@zelixir.com; Weifeng Li, School of Physics, Shandong University, Jinan, Shandong, 250100, China. E-mail: lwf@sdu.edu.cn.

**Details of additional evidence for generalization capability**

(i) Protein similarity test and (ii) ligand similarity test

We carried out a comprehensive evaluation of dataset overlap. Since HybridSP is composed of three statistical potential components, and because the original training code for KORP-PL and ITScoreAff is not publicly available, we were unable to perform similarity-sensitivity tests for those two components. Therefore, we only evaluated DrugResidue$_W$.

The results show that when the similarity between the training set and CASF-2016 protein/ligand components is restricted to below 0.5, model performance decreases. However, this decline may partly result from the substantial reduction in data size. To further examine this issue, we also evaluated the model on the full general set of PDBbind 2020, which contains 19,443 complexes, without filtering by resolution. Under the same similarity cutoff, model6 generally outperforms model1, indicating that dataset size itself can have a substantial influence on the learned statistical distribution. We do not deny that similarity between training and test

samples may bias a model toward certain cases. However, in the scoring tasks of binding poses, even deep learning methods are generally not subjected to strict similarity-based deduplication. A common practice is to remove only complexes that are exactly identical between the training and test sets [1-3]. This is reasonable because even the same protein may adopt different binding poses when complexed with different ligands. Therefore, although we did not apply strict similarity-based deduplication, our comparisons with different deep learning methods remain fair.

Table S1: Performance of DrugResiduew on the CASF-2016 benchmark across different similarity thresholds between training and test sets. seq_simi denotes the sequence similarity between proteins in the training set and those in CASF-2016; lig_simi denotes the fingerprint similarity of ligand molecules.

| Model | Training set | size | Scoring Power | Ranking Power | Docking Power (w/o ligand) | Docking Power (with ligand) | Screening Power (EF) | Screening Power (success rate) |
|---|---|---|---|---|---|---|---|---|
| 0 | resolution < 2.5; remove PDB entry from CASF-2016 and Unbias-2019 | 15145 | 0.389 | 0.493 | 80.4 | 87.0 | 11.02 | 24.6 |
| 1 | resolution < 2.5; remove PDB entry from CASF-2016 and Unbias-2019; seq_simi < 0.5 | 10749 | 0.361 | 0.423 | 76.8 | 84.2 | 9.49 | 21.1 |
| 2 | resolution < 2.5; remove PDB entry from CASF-2016 and Unbias-2019; lig_simi < 0.5 | 12947 | 0.378 | 0.458 | 80.0 | 84.6 | 10.14 | 22.8 |
| 3 | resolution < 2.5; remove PDB entry from CASF-2016 and Unbias-2019; seq_simi < 0.5 and lig_simi < 0.5 | 9510 | 0.347 | 0.428 | 76.1 | 83.2 | 9.45 | 21.1 |
| 4 | resolution < 2.5; remove PDB entry from CASF-2016 and Unbias-2019; seq_simi < 0.5 or lig_simi < 0.5 | 14186 | 0.388 | 0.444 | 81.4 | 87.0 | 10.2 | 22.8 |
| 5 | all general set of PDBbind 2020 | 19443 | 0.426 | 0.560 | 80.7 | 88.1 | 12.03 | 28.1 |
| 6 | seq_simi < 0.5 | 14302 | 0.379 | 0.444 | 78.6 | 85.3 | 10.53 | 22.8 |
| 7 | lig_simi < 0.5 | 16500 | 0.392 | 0.437 | 78.9 | 84.6 | 12.01 | 28.1 |
| 8 | seq_simi < 0.5 and lig_simi < 0.5 | 12666 | 0.367 | 0.440 | 77.2 | 83.2 | 10.27 | 22.8 |
| 9 | seq_simi < 0.5 or lig_simi < 0.5 | 18136 | 0.404 | 0.454 | 80.7 | 86.7 | 11.83 | 28.1 |

(iii) Cross-docking

We evaluated cross-docking performance on the 3D-Disco dataset and compared HybridSP with RTMScore. The workflow first uses AutoDock Vina to generate 20 (at most) docking poses and then applies HybridSP or RTMScore for rescoring. Among all 4,484 cases, the fraction of docking runs with minimum RMSD < 2 Å was only 48.02%, which sets the upper bound of the achievable success rate. In addition, more than 1,000 complexes could not be successfully scored by RTMScore. To ensure a fair comparison, we recalculated the success rate using the 3,468 cases for which RTMScore produced valid outputs. Under this setting, HybridSP achieves an accuracy only slightly lower than RTMScore by less than 2%. Both methods substantially outperform the raw Vina scoring results. Moreover, when the top five ranked poses are considered, the success rate approaches the maximum possible value, indicating that near-native conformations can be identified almost completely.

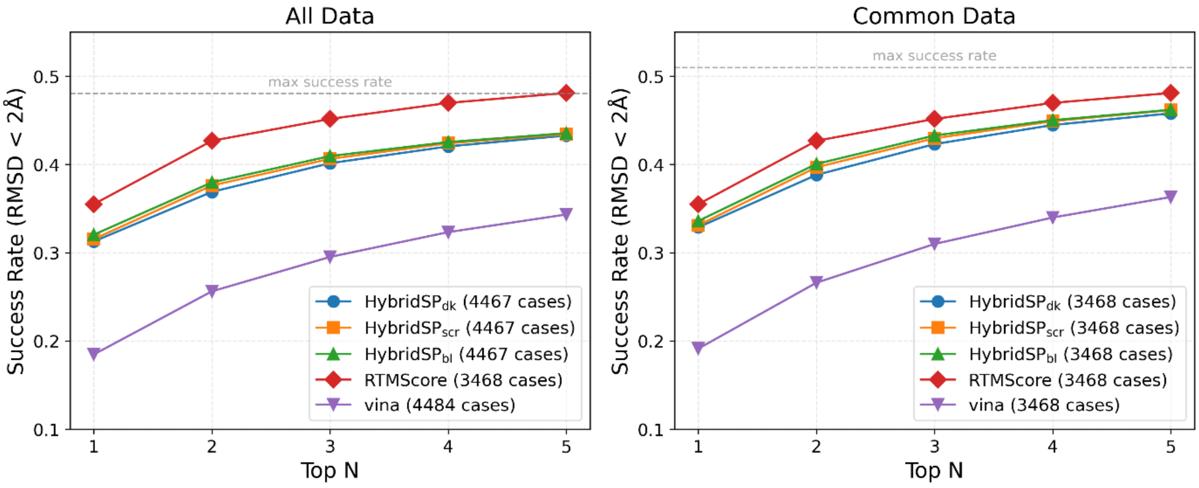


**Figure S1:** Success rates on the 3D-Disco cross-docking dataset. The left panel shows the success rates for each method across all successfully predicted cases for that method, respectively. The right panel shows the success rates evaluated on the intersection of cases where all methods produced an output.

(iv) Semi-prospective screening on CBL-B and CHD1

We further performed semi-prospective screening on two targets, CBL-B and CHD1, using recently published studies in 2024 that have available crystal structures, in order to evaluate the performance of the model in realistic scenarios. Binding affinity data associated with these two

targets were collected from ChEMBL and BindingDB. Compounds with Kd (or IC50) < 1 μM were treated as true positives (active); otherwise, they were treated as true negatives (inactive). To better reflect real-world screening conditions, we additionally sampled negative compounds from ZINC at a ratio of 500 negatives per positive, which is ten times higher than the 1:50 ratio commonly used in DUD-E. When selecting inactive compounds, we attempted to match the molecular weight of each negative compound to that of the corresponding positive compound as closely as possible. The final datasets contained 780 (777 molecules were successfully docked by Vina) positive compounds and 390,048 (389,855 molecules were successfully docked by Vina) negative compounds for CBL-B, and 29 positive compounds and 14,528 (14,523 molecules were successfully docked by Vina) negative compounds for CHD1. The results show that the model achieves very strong enrichment on both targets. Among the top 100 compounds ranked by the model, nearly 20 are true positives. For CBL-B, most true negatives are ranked beyond 10,000. For CHD1, although the negative compounds are ranked relatively higher, $HybridSP_{scr}$ and $HybridSP_{bl}$ still place most of them beyond the top 100. These two case studies demonstrate that HybridSP has strong generalization capability in realistic applications. In comparison, RTMScore performs poorly on CBL-B and reasonably on CHD1, but its reported EF values are not fully reflective of the true situation, because it suffers from a very high failure rate during scoring, resulting in an effective positive-to-negative ratio that is not 1:500. Specifically, in the CBL-B case, 178,300 out of 389,855 negative compounds failed to be scored; in the CHD1 case, 6,591 out of 14,523 negative compounds failed to be scored. The active and inactive molecules and the screening results of these two targets are available at https://github.com/zelixirSH/HybridSP.

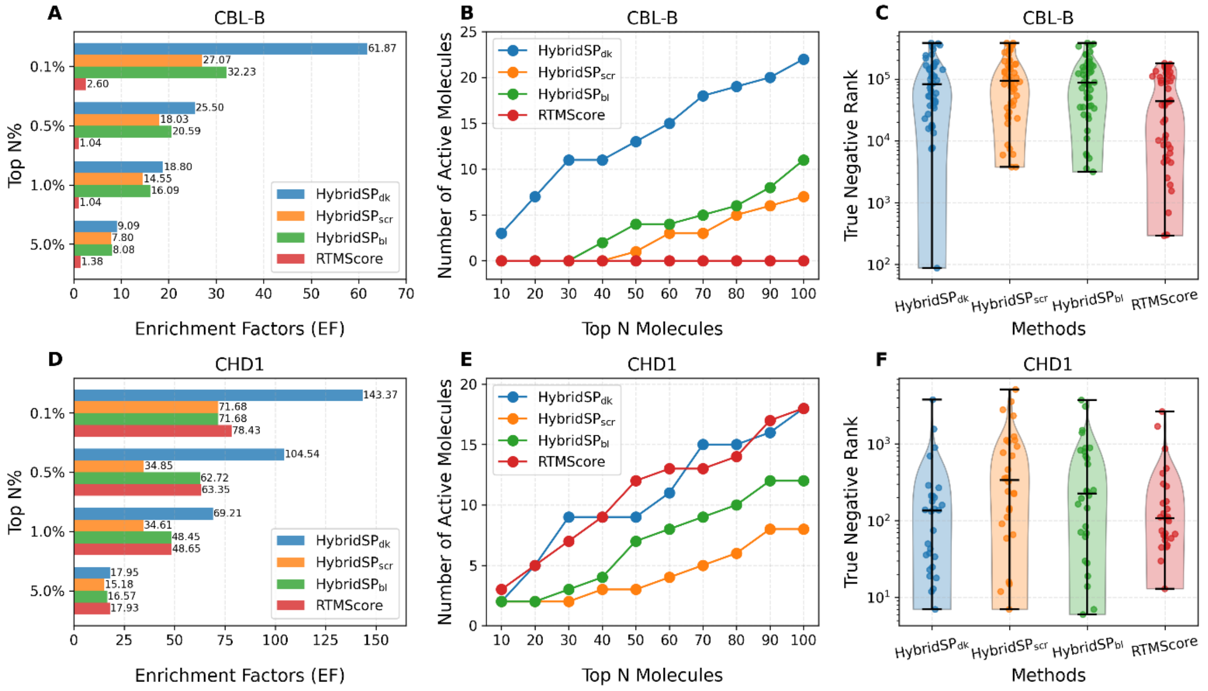


Figure S2: Virtual screening results on targets CBL-B (A-C) and CHD1 (D-F). (A, D) Enrichment factors. (B, E) Number of true positive molecules among the top 100 ranked compounds. (C, F) Ranking of true negative molecules.

## References


1. Shen C, Zhang X, Hsieh C-Y *et al.* A generalized protein–ligand scoring framework with balanced scoring, docking, ranking and screening powers. *Chem Sci* 2023;14:8129–46. https://doi.org/10.1039/d3sc02044d
2. Shen C, Zhang X, Deng Y *et al.* Boosting protein–ligand binding pose prediction and virtual screening based on residue–atom distance likelihood potential and graph transformer. *J Med Chem* 2022;65:10691–706. https://doi.org/10.1021/acs.jmedchem.2c00991
3. Wang Z, Wang S, Li Y *et al.* A new paradigm for applying deep learning to protein–ligand interaction prediction. *Brief Bioinform* 2024;25:bbae145. https://doi.org/10.1093/bib/bbae145
